# Effect of neurofeedback-assisted mindfulness training on migraine severity, associated disability, sleep quality, and emotional distress: protocol for a randomized controlled trial

**DOI:** 10.3389/fdgth.2026.1962535

**Published:** 2026-10-26

**Authors:** Chaimae Zahri-Haidar, Irene A. Garcia-Yu, Nerea Sanchez-Sanchez, Jesús Gonzalez-Sanchez, Hsin-Chien Lee, Victoria Alejos-Herrera, Virginia Iglesias-Sierra, Hsueh-Chen Lu, Jose I. Recio-Rodriguez

**Affiliations:** 1 Facultad de Enfermería y Fisioterapia, Universidad de Salamanca, Salamanca, Spain; 2 Facultad de Medicina, Universidad de Salamanca, Instituto de Investigación Biomédica de Salamanca (IBSAL), Salamanca, Spain; 3 Facultad de Enfermería y Fisioterapia, Universidad de Salamanca, Instituto de Investigación Biomédica de Salamanca (IBSAL), Complejo Asistencial Universitario de Salamanca (CAUSA), Salamanca, Spain; 4 Facultad de Enfermería y Fisioterapia, Universidad de Salamanca, Instituto de Investigación Biomédica de Salamanca (IBSAL), Salamanca, Spain; 5 College of Humanities and Social Sciences, Graduate Institute of Humanities in Medicine, Taipei Medical University, Taipei, Taiwan; 6 Complejo Asistencial Universitario de Salamanca (CAUSA), Instituto de Investigación Biomédica de Salamanca (IBSAL), Salamanca, Spain; 7 Gerencia de Atención Primaria de Zamora, Instituto de Investigación Biomédica de Salamanca (IBSAL), Salamanca, Spain; 8 College of Medicine, Graduate Institute of Clinical Psychology, Taipei Medical University, Taipei, Taiwan; 9 Facultad de Enfermería y Fisioterapia, Universidad de Salamanca, Instituto de Investigación Biomédica de Salamanca (IBSAL), Red de Investigación en Cronicidad, Atención Primaria y Promoción de la Salud (RICAPPS), Salamanca, Spain

**Keywords:** migraine, migraine disorders, mindfulness, neurofeedback, randomized controlled trial

## Abstract

**Background:**

Migraine is a highly prevalent neurological disorder and a leading cause of worldwide disability, exerting significant clinical, social, and economic burdens. While pharmacological options exist, their efficacy is often limited by tolerability issues and risk of medication-overuse headache. Neurofeedback (NF) combined with mindfulness training has emerged as a promising non-pharmacological neuromodulation technique. However, clinical evidence in migraine management remains limited by methodological heterogeneity and small sample sizes. This study protocol aims to evaluate the efficacy of a Neurofeedback-Assisted Mindfulness Training Program (NAMTP) in adults with medium-to-high-frequency episodic migraine.

**Methods/design:**

This is a randomized, parallel-group, controlled clinical trial protocol. Sixty-six (*N* = 66) adults diagnosed with medium-to-high-frequency episodic migraine (5–14 monthly migraine days) will be recruited and randomized (1:1 ratio) into either the Intervention Group (IG: standard care plus NAMTP) or the Control Group (CG: standard routine care alone). The IG will undergo a 12-week home-based program consisting of 60 sessions (15–20 min each, 5 times per week) of mindfulness training assisted by the Muse® electroencephalography (EEG) headband. Assessments will be conducted at baseline, 12 weeks (post-intervention), and 24 weeks post-randomization. The powered primary outcome is the change in Monthly Migraine Days (MMDs). Secondary outcomes include migraine-associated disability (MIDAS), headache impact (HIT-6), sleep parameters (actigraphy and PSQI), quality of life (MSQ v.2), and mood symptoms (BDI-II and STAI-Y).

**Discussion:**

This protocol addresses the urgent need for scalable, non-pharmacological therapies in migraine management. By combining objective neurophysiological monitoring with behavioral training within a rigorous randomized controlled framework, this trial will clarify whether NAMTP can reduce headache burden, optimize sleep patterns, and diminish affective distress. The findings are expected to provide standardized, actionable evidence regarding the integration of digital neurofeedback tools into routine clinical care for episodic migraine.

**Clinical Trial Registration:** ClinicalTrials.gov NCT07661888.

## 1 Introduction

Migraine constitutes one of the neurological diseases with the greatest impact on global public health, currently considered the second leading cause of disability worldwide and the first among women aged 15–49 years (1). Its high prevalence, coupled with the recurrent and disabling nature of the episodes, makes it a health problem of great clinical, social, and economic relevance. In Spain, population-based studies estimate a prevalence close to 12.6%, further observing a marked difference by sex, with a significantly higher burden in women than in men (17.2% compared to 8.0%) (2). These figures are consistent with recent international epidemiological estimates, which place the global prevalence at around 14.0%, affecting approximately 15.5% of women and 7.4% of men (3).

Beyond the frequency of attacks, migraine is associated with a considerable clinical burden and a broad spectrum of vascular and psychiatric comorbidities. Migraine increases the risk of vascular disorders, both at the cerebral level—such as ischemic and hemorrhagic stroke (4, 5), and subclinical brain lesions (6),—and in other areas of the body, including ischemic heart disease and peripheral artery disease (7). In parallel, there is a high coexistence with psychological and psychiatric disorders, including depression, anxiety, bipolar disorder, attention deficit disorder, and an increased suicide risk (8).

The functional impact of migraine extends substantially to quality of life, psychosocial functioning, and workplace productivity. The increase in absenteeism and the decrease in work performance show a direct relationship with the increased number of headache days per month, constituting one of the main factors of disability associated with the disease (1, 9). Furthermore, migraine is the most important reason for visiting healthcare services among those affected by this condition, accounting for 66.2% of healthcare center visits for these individuals (9). In economic terms, suffering from migraine is associated in Spain with an approximate 1.7-fold increase in healthcare costs per patient (10).

Migraine also exerts a significant impact on multiple dimensions of daily functioning, including interpersonal relationships, social participation, and emotional well-being (11). Likewise, a significant impairment in health-related quality of life and relevant alterations in sleep architecture and quality have been documented (12, 13).

Despite advances in pharmacological treatment, these options present limitations related to efficacy, tolerability, contraindications, and adverse effects (14, 15). Likewise, the inappropriate use of medication can lead to medication-overuse headache, increasing the burden of the disease (16). In this context, interest in non-pharmacological interventions has grown, especially those based on neuromodulationn (17).

Among neuromodulation-based interventions, neurofeedback (NF) has emerged as a promising technique. NF is a form of biofeedback training that utilizes the recording of brain activity through imaging techniques to achieve, via a feedback process, the control and regulation of brain activity patterns (18). Based on the principles of operant conditioning, patients gradually learn through the positive reinforcement provided by the feedback. Real-time information on brain activity is provided by imaging techniques such as brain magnetic resonance imaging (MRI), functional near-infrared spectroscopy (fNIRS), or electroencephalography (EEG), with the latter being the most widely used in NF due to its lower cost, accessibility, and ease of clinical implementation (18).

In this context, mindfulness-based interventions have gained attention in headache management. However, evidence regarding their specific effect on migraine frequency remains nuanced. In a benchmark active-controlled randomized trial, Wells et al. (19) demonstrated that Mindfulness-Based Stress Reduction (MBSR) did not reduce monthly migraine frequency significantly more than enhanced headache education. Nevertheless, MBSR yielded meaningful improvements in migraine-related disability, health-related quality of life, self-efficacy, pain catastrophizing, and depressive symptoms. This distinction highlights that while mindfulness primarily mitigates the affective and functional burden of migraine, combining it with real-time EEG neurofeedback (NAMTP) represents an unexplored strategy to potentially enhance behavioral self-regulation and optimize both primary clinical frequency and secondary patient-reported outcomes. Concurrently, Neurofeedback-Assisted Mindfulness Training interventions have shown promising results in modulating psychological well-being and emotional self-regulation, with evidence of reductions in perceived stress, anxiety, depression, fatigue, and insomnia, as well as improvements in resilience and neurophysiological parameters associated with a deeper state of relaxation measured via EEG (20–22). In the field of migraine, Golshan et al. (15), in a randomized clinical trial with EEG-based neurofeedback, observed a significant improvement in self-efficacy for disease management, although no clear benefits were demonstrated regarding disability or attack severity compared to the control group.

Despite its biological rationale and the success of past experiences in reducing both the frequency and severity of migraine episodes, a greater number of clinical trials with control groups and homogeneous protocols are needed, given the growing increase in the number of NF devices, the reduction of their costs, and the possibility for patients to undergo this therapy autonomously in some cases.

In this context, the primary objective of this trial is to evaluate the clinical effectiveness of the complete Neurofeedback-Augmented Mindfulness Training Program (NAMTP)—a multimodal digital intervention combining mindfulness, EEG-contingent feedback, and app-based monitoring—as an add-on to usual care compared with usual care alone in reducing Monthly Migraine Days (MMDs) in adults with medium-to-high-frequency episodic migraine. Secondary objectives include assessing changes in headache-related disability, quality of life, acute medication consumption, and overall intervention feasibility.

## 2 Methods and analysis

### 2.1 Design

The design of this study corresponds to a randomized, parallel-group, controlled clinical trial. The study was registered at ClinicalTrials.gov (Identifier: NCT07661888) on June 22, 2026. This protocol has been drafted in accordance with the SPIRIT (Standard Protocol Items: Recommendations for Interventional Trials) 2025 Statement guidelines (23).

### 2.2 Participants and recruitment

Study participants will be recruited from the outpatient clinics of the Neurology Department at the University Hospital of Salamanca and the Hospital of Ávila (Spain). Subject inclusion will be performed through consecutive sampling according to ICHD-3 diagnostic criteria and guidelines established by the International Headache Society (IHS) for clinical trials in migraine (16, 24).

Inclusion criteria will be: (1) Adults aged ≥18 years; (2) Established clinical diagnosis of episodic migraine, with or without aura, according to the ICHD-3 criteria (Code 1.1 or 1.2); (3) Medium-to-high-frequency episodic migraine, defined strictly as having 5–14 Monthly Migraine Days (MMDs) and <15 total Monthly Headache Days (MHDs) per month during the prospective baseline period; (4) High compliance (≥80%) during the 4-week prospective baseline headache diary period; (5) Voluntary agreement to participate and signing of informed consent (16).

Exclusion criteria will be: (1) Chronic Migraine (CM): Fulfilling ICHD-3 criteria for chronic migraine (Code 1.3), defined as headache occurring on ≥15 days per month for >3 months, with at least 8 of those days meeting migraine criteria; (2) Medication-Overuse Headache (MOH): Fulfilling ICHD-3 criteria for MOH (Code 8.2), defined as headache occurring on ≥15 days per month in a patient with pre-existing headache disorder, accompanied by regular overuse of acute medication for >3 months (≥10 days/month for triptans, opioids, or combination analgesics; or ≥15 days/month for simple analgesics/NSAIDs); (3) History of severe psychiatric disorders requiring treatment within the past 6 months (e.g., psychosis, bipolar disorder) or severe cognitive impairment; (4) Structural brain lesions or medical conditions contraindicating EEG headband use or neurofeedback training (16).

Prospective Baseline Period: Following initial screening, candidates will enter a 4-week (28-day) prospective run-in baseline period. Participants will complete a standardized daily electronic or paper headache diary recording headache occurrence, duration (hours), peak pain intensity (0–10 VAS), associated symptoms (nausea, photophobia, phonophobia), and type/dose of acute rescue medication taken. Only participants who maintain ≥80% diary completion compliance (≥22.5 logged days) and whose prospectively collected data confirm 5–14 MMDs (and confirm the absence of CM and MOH) will be eligible for baseline assessment and subsequent 1:1 randomization.

The sample size calculation was performed using Epidat 4.2 software. Assuming an alpha risk of 0.05 and a statistical power greater than 0.80 in a two-tailed test, 33 subjects are required in the experimental group and 33 in the control group to detect a difference of 3.6 or more migraine days in the last month between the two groups. This target difference was estimated based on clinical trial data by Wang et al. (25) and aligns with or exceeds the Minimal Clinically Important Difference (MCID) established in episodic migraine trials (24). A common standard deviation of 4.8 days is assumed, alongside an estimated follow-up loss rate of 15%.

### 2.3 Inclusion and randomization procedure

Following initial contact in the outpatient clinics of the Neurology Department at the University Hospital of Salamanca and Hospital of Ávila (Spain), an in-person visit will be scheduled. During this visit, one of the study investigators will present the study, address any questions, and proceed with the signing of the informed consent form.

After verifying that the participant meets the inclusion criteria, a baseline assessment will be conducted to collect clinical, sociodemographic, and study outcome variables. Subsequently, participants will be randomly assigned (1:1 ratio) to one of the two study arms: the Intervention Group [IG: Neurofeedback-Assisted Mindfulness Training Program (NAMTP) + Standard Care] or the Control Group (CG: Standard Care alone). Randomization will be performed using Epidat 4.2 software, with a 1:1 allocation ratio using permuted block randomization with variable block sizes (4 and 6) to ensure balanced group distribution. Allocation will remain concealed until the inclusion of the participants.

The investigator responsible for generating the randomization sequence will not participate in data collection or outcome assessment. Likewise, the investigators responsible for follow-up assessments will be blinded to the participants' group allocation throughout the entire study. Participants will be instructed not to disclose their group allocation to the evaluators during follow-up visits. Statistical analysis of the data will also be performed blindly with respect to the allocation of participants to the intervention groups.

An assessment will be conducted at 12 weeks, and a follow-up assessment will be performed at 24 weeks post-randomization for all participants, during which clinical, sociodemographic, and study outcome variables will be re-evaluated (Figure 1).

**Figure 1:**
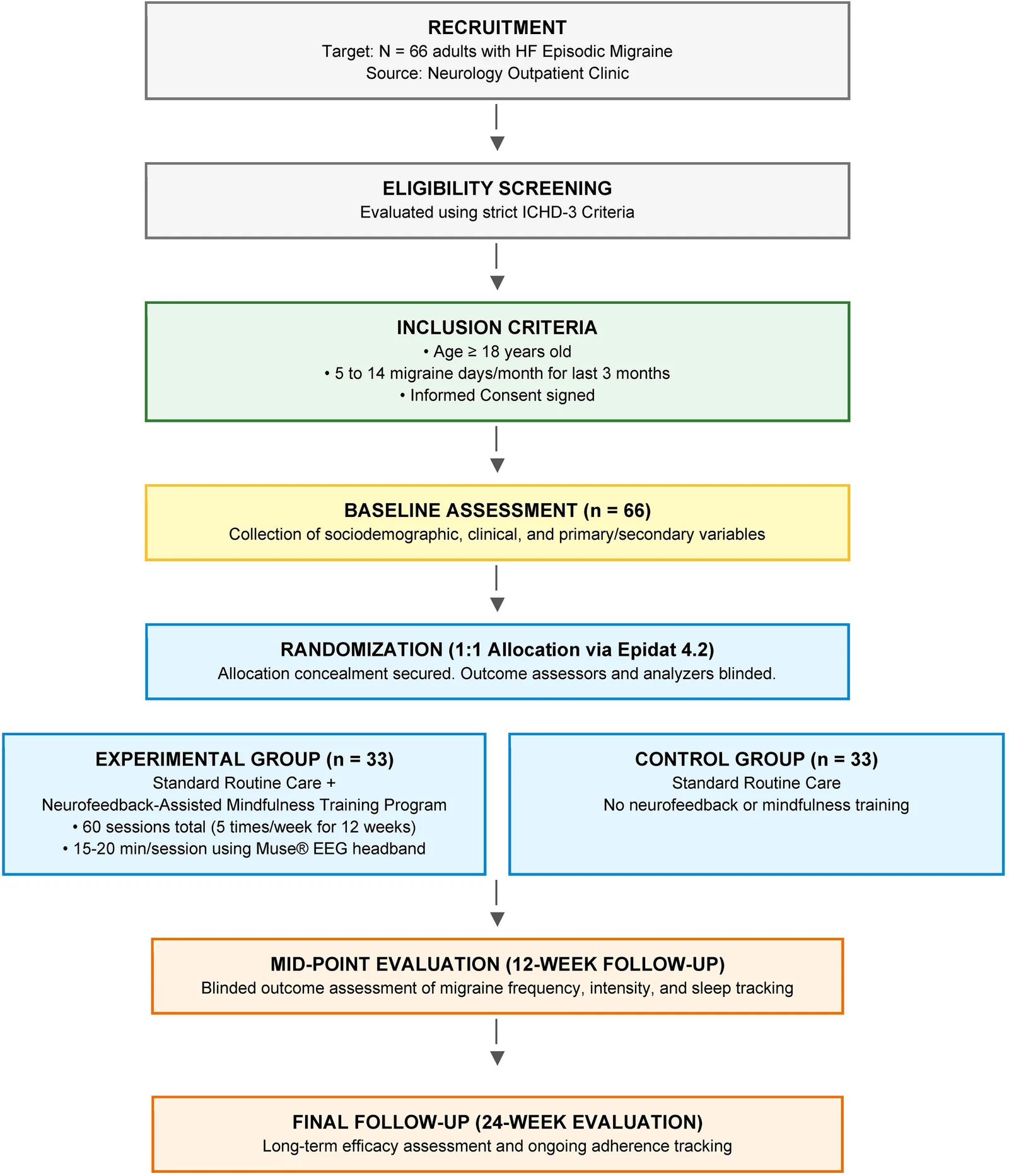
Study protocol flowchart.

### 2.4 Description of the intervention

Throughout the study, participants in both groups will continue to receive their Standard Routine Care. To prevent pharmacological confounding, candidates using preventive migraine medication must have been on a stable regimen and dosage for at least 12 weeks (3 months) prior to entering the prospective baseline period, and will be instructed to keep this regimen unchanged throughout the 24-week trial period unless clinically mandatory. All concurrent acute and preventive medication use, including any dose adjustments or regimen changes, will be systematically logged at every follow-up milestone (12 and 24 weeks).

In addition to this baseline care, participants assigned to the Intervention Group (IG) will undergo a complementary attentional training and meditation program assisted by neurofeedback using the Muse headband, whereas those assigned to the Control Group (CG) will continue exclusively with their routine care, receiving neither the device-assisted intervention nor any additional meditation guidelines during the intervention period.

The IG will follow a 60-session (NAMTP) program over a 3-month period, conducted at a frequency of 5 times per week. Each session will last between 15 and 20 min. Participants will perform these daily sessions at home in a quiet, private room, instructed to adopt a comfortable seated position with their eyes closed during the neurofeedback-assisted mindfulness practice.

The number and duration of the sessions have been established using previous studies that employed comparable methodologies based on brief, guided mindfulness interventions as a reference. In those studies, clinically significant improvements were described after 20 sessions (26, 27), which provided a basis for selecting a similar intervention schedule for this study.

#### 2.4.1 Muse® device

The instrument used for the NAMTP sessions will be the Muse® device (InteraXon Inc.), a surface EEG-based interface designed for real-time monitoring of cortical activity and cognitive self-regulation training through NF. More information can be found at: https://choosemuse.com/.

The study will utilize the Muse 2 and Muse S Headband models (Figure 2), two technically equivalent headband designs as they both share the same sensors. The decision to include both models is based on their technical equivalency and on maximizing device availability and resource utilization for trial execution. However, they differ in design and ergonomics; the former is a rigid headband, while the latter is an elastic fabric band that, additionally, allows for sleep monitoring.

**Figure 2:**
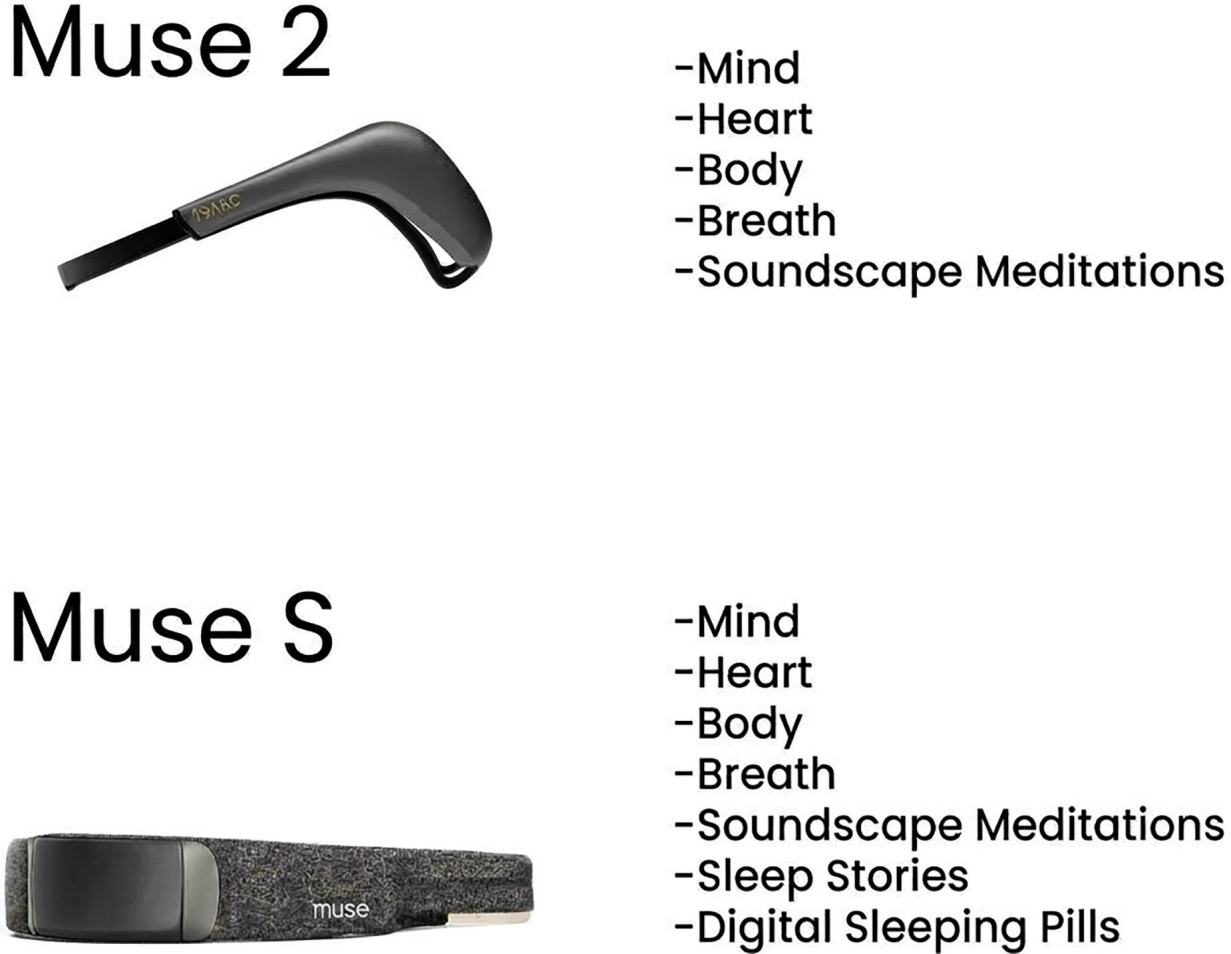
Types of muse headbands used in the study. Reprinted with permission from Muse 2 and Muse S Headbands Product Overview by Interaxon Inc., © Muse by Interaxon Inc., Interaxon Inc. (choosemuse.com).

Muse devices feature multiple sensors that have been tested and validated against standard EEG systems, providing real-time feedback on four biological parameters: brainwaves (EEG), heart rate (via photoplethysmography), posture and movement (accelerometer), and respiration.

The headbands integrate seven strategically placed dry conductive sensors, based on the International 10–20 system for electrode placement, to record EEG signals from four channels. Two are located on each side of the forehead (AF7 and AF8) over the prefrontal cortex, two are in temporal positions (TP9 and TP10) in the regions posterior to the pinnae, one active reference sensor is located in the mid-frontal area (Fpz), and the remaining two auxiliary ground/reference noise-reduction electrodes are also positioned on the forehead to optimize signal quality (28, 29). Figure 3 shows the sensor locations on each headband model.

**Figure 3:**
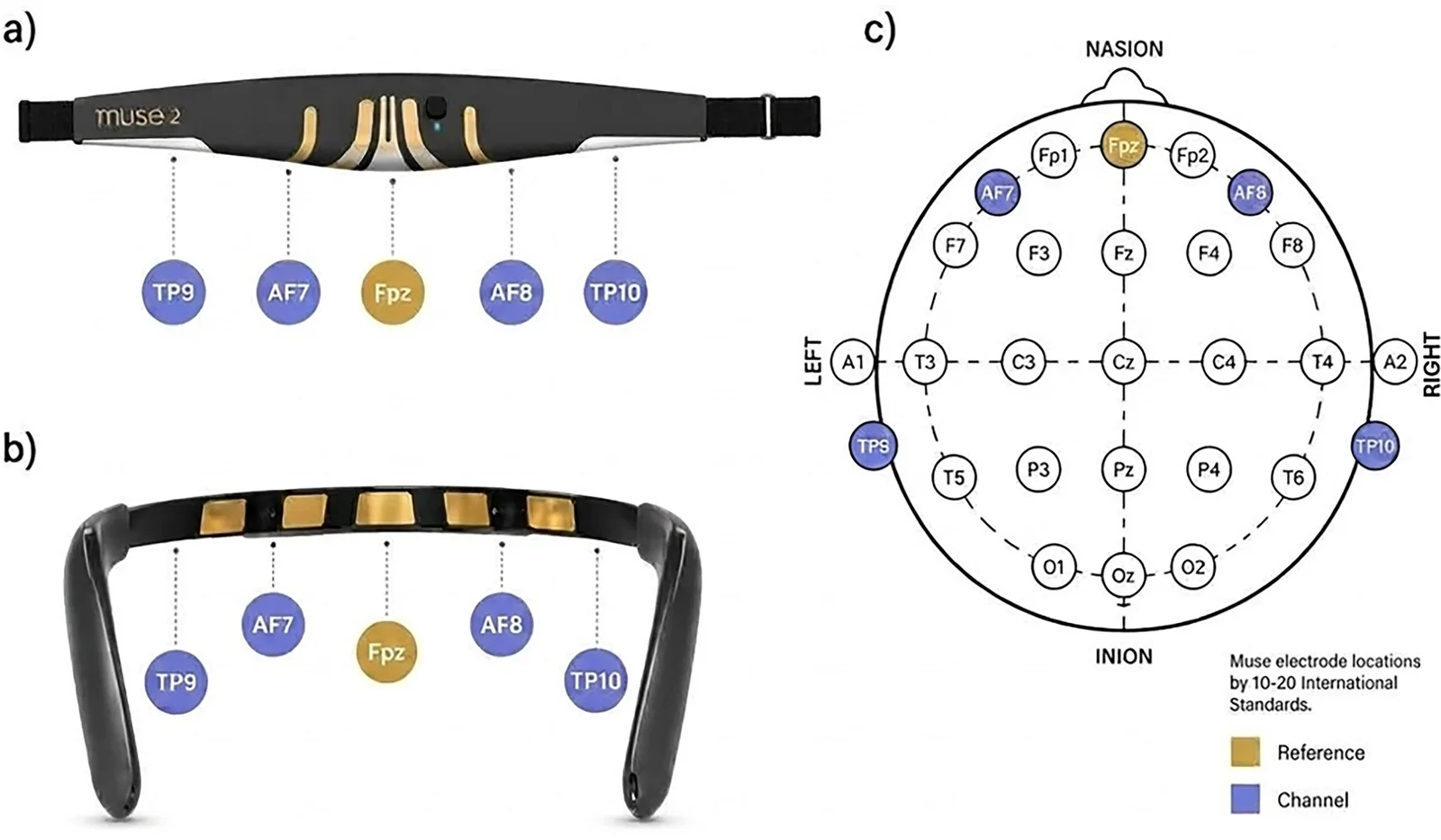
Electrode distribution with the muse S **(a)** and muse 2 **(b)** headbands, and their position on the cerebral cortex **(c)**. Reprinted with permission from Muse 2 and Muse S Headbands Product Overview by Interaxon Inc., © Muse by Interaxon Inc., Interaxon Inc. (choosemuse.com).

Through its sensors, the device records brain signals and transmits them via Bluetooth to the Muse: EEG Meditation & Sleep mobile application, where they undergo advanced mathematical processing that utilizes machine learning algorithms to analyze the different brainwaves.

The algorithm processes brain activity in the delta (1–4 Hz), theta (4–8 Hz), alpha (8–13 Hz), beta (13–30 Hz) and gamma (30–44 Hz) frequency bands to estimate the participant's mental state (29). The Muse algorithm analyzes the power ratio and stability between the waves to categorize EEG activity into three states: calm, active, or neutral. The calm state is characterized by a rhythmic and stable signal and a predominance of alpha waves.

Previous studies based on Muse® have established these markers as reliable behavioral correlates of attentional control and mindfulness engagement, showing reductions in mind-wandering (30, 31).

The device provides an auditory neurofeedback mechanism. When the device detects calm states, it provides positive reinforcement in the form of pleasant, harmonic, and soft sounds; conversely, when it identifies an increase in beta activity associated with a loss of attentional focus, it delivers corrective feedback in the form of more intense sounds to serve as a cue for the individual to redirect their attention. During the baseline onboarding session, participants are explicitly educated on these auditory feedback mechanics (e.g., quiet weather and bird sounds indicate a calm mental state, whereas intensified wind sounds signal mind-wandering and prompt a gentle redirection of attention).

#### 2.4.2 Session scheduling

During the first sessions of the intervention, two pre-established programs from the application will be used, aimed at beginner meditation users (Discover Mind Biofeedback and Muse Essentials). These programs are designed for progressive initiation into meditation practices and NAMTP.

Starting from the third session, unguided “Browse by Goal” sessions will be utilized, aimed at improving stress coping, elevating performance, and improving mind and posture. The session schedule followed is detailed in Supplementary Table S1.

#### 2.4.3 Session recording

Once each session is completed, the application will generate a report with the training results, including: session duration, time spent in each mental state (with time in calm considered the primary indicator of the session), and complementary data such as heart rate and body posture. Among the recorded parameters, key metrics include “recoveries” (defined as the number of times the user managed to restore attentional focus after an episode of mind-wandering) and “birds” (which represent periods of sustained attention exceeding 5 s).

Participants will be required to complete a paper-based log form following the completion of each session—as detailed in Supplementary Table S2—using the data from the report. Adherence to the intervention will be continuously monitored and quantified through these individual session reports, tracking both total completed sessions (out of the 60 prescribed) and weekly practice frequency. Adherence to the intervention will be monitored by tracking these individual session reports.

### 2.5 Outcome measures

#### 2.5.1 Main outcome

##### 2.5.1.1 Migraine frequency and severity

The primary powered outcome is strictly defined as the change in Monthly Migraine Days (MMDs), recorded prospectively via a continuous daily headache diary during each 28-day assessment window (baseline, 12 weeks post-randomization, and 24 weeks follow-up). Additionally, headache frequency will be tracked using Monthly Headache Days (MHDs) and discrete migraine attacks, alongside the frequency (days/month) and dosage of acute rescue medication use. Pain characteristics, including location, duration, and associated symptoms, will also be collected using the same daily tracking method.

Headache severity will be rated daily using a 0–100 mm Visual Analog Scale (VAS, where 0 = no pain and 100 = maximum imaginable pain) and a 4-point categorical severity scale (0 = none, 1 = mild, 2 = moderate, 3 = severe). Monthly pain severity will be summarized as the mean daily peak VAS score calculated across all reported headache days within each 28-day assessment period. Additionally, overall monthly pain burden will be summarized as the monthly cumulative pain index [calculated as the sum of (headache duration in hours × daily peak VAS score)/100] to capture both duration and intensity dynamics across assessment milestones.

#### 2.5.2 Secondary outcomes

##### 2.5.2.1 Disability and impact on daily life

The validated Spanish version of the Migraine Disability Assessment (MIDAS) questionnaire will be administered (32). The MIDAS is a self-administered instrument widely used in clinical practice and research to determine the degree of migraine-induced disability in adults. The questionnaire consists of seven items in total. The first five items make up the main scale and evaluate the number of days the patient has missed or experienced a significant reduction in performance due to headache across three essential areas of daily life: work or school productivity, household chores, and social, family, or leisure activities. The remaining two items (the sixth and seventh) are not added to the disability score; instead, they serve to gather complementary information regarding the overall frequency and intensity of the headache experienced by the subject. The overall score of the questionnaire is obtained by summing exclusively the days reported in the first five questions, ranging from 0 to a theoretical maximum of 90 days or more (as it assesses the cumulative impact over the past 3 months). To interpret this overall score, four standardized cut-off points are used to classify the patient's degree of disability as follows: Grade I (0–5 points) indicates little or no disability; Grade II (6–10 points) represents mild disability; Grade III (11–20 points) corresponds to moderate disability; and Grade IV (21 points or more) indicates severe disability. Items 6 and 7, referring solely to clinical frequency and intensity, are analyzed independently and are neither calculated within this range nor do they affect the disability cut-off points.

Additionally, the Headache Impact Test (HIT-6) will be administered (33) whose Spanish version retains the same psychometric properties as the English version (34). The HIT-6 is a self-administered instrument designed to measure and assess the overall impact that headache and migraine have on the patient's daily life. The questionnaire consists of six items covering various dimensions of daily functioning and quality of life: physical pain, social functioning, role functioning, vitality, cognitive functioning, and associated psychological distress. Each of the six questions offers five behavioral response options based on the frequency of the experienced impact: “Never” (6 points), “Rarely” (8 points), “Sometimes” (10 points), “Very often” (11 points), and “Always” (13 points). The overall score of the questionnaire is obtained by summing the scores assigned to each response, ranging from a minimum of 36 points to a maximum of 78 points. To interpret this result, four standardized cut-off points are used to classify the degree of impact on the subject's life: a score of 49 points or less indicates little or no impact; 50–55 points represents mild impact; 56–59 points corresponds to moderate impact; and 60 points or more indicates a severe impact on the individual's daily functioning.

##### 2.5.2.2 Sleep quality

Sleep quality will be measured using accelerometry (ActiGraph). This system continuously captures accurate and reliable sleep/wake information, circadian rhythms, and physical activity, providing detailed reports and sleep/wake graphs, and calculating sleep statistics such as sleep onset, sleep latency, sleep duration, and sleep efficiency using various validated scoring algorithms. The ActiGraph system utilizes a wrist-worn accelerometer to measure total sleep time (TST), sleep efficiency, and wake after sleep onset (WASO). The ActiGraph GT3X + model (ActiGraph®, Pensacola, FL, USA) will be used. The ActiGraph GT3X+ has been previously validated and is considered a reliable method for estimating sleep characteristics in both adult and adolescent populations (35). All sleep parameters will be determined using ActiLife 6 software (Version 6.13.1, ActiGraph, Pensacola, FL, USA). Actigraphy data will be collected over a period of 5 consecutive days. Actigraphs (ActiGraph GT3X+) will be handed to participants at the end of each assessment visit with verbal and written instructions to wear the device continuously on the non-dominant wrist for 5 consecutive days and nights (removing it only during prolonged water submersion). Raw data will be retrieved via ActiLife software upon device return at the clinic.

Additionally, the Pittsburgh Sleep Quality Index (PSQI) will be administered. This is a valid and highly reliable instrument specifically designed to measure sleep quality (36). It consists of 19 questions that generate seven main components. Each component is scored from 0 to 3 points, where a lower score indicates no problems, whereas a higher score denotes worsening problems in the following order: (1) subjective sleep quality (very good vs. very bad), (2) sleep latency (≤15 min to >60 min), (3) sleep duration (≥7 h to <5 h), (4) sleep efficiency (≥ 85% to <65% hours of sleep/hours in bed), (5) sleep disturbances (not during the past month to ≥3 times per week), (6) use of sleep medication (none to ≥3 times per week), and (7) daytime dysfunction (not a problem to a very big problem). The scores of the seven components are then summed to yield a global score ranging from 0 to 21 points.

##### 2.5.2.3 Quality of life

Quality of life will be analyzed using a migraine-specific quality of life instrument: the Migraine-Specific Quality of Life Questionnaire (MSQ) v.2. The questionnaire consists of 14 items with six response options ranging from “None of the time” to “All of the time”. Each of the three domains of the MSQ is scored independently on a linearly transformed scale from 0 to 100, in which a higher score indicates a better health status (37).

##### 2.5.2.4 Symptoms of anxiety and depression

The Beck Depression Inventory-II (BDI-II) and the State-Trait Anxiety Inventory (STAI-Y) will be used. The Beck Depression Inventory-II (BDI-II) is a widely used 21-item self-administered instrument designed to assess the presence and severity of depressive symptomatology in adults and adolescents, both in clinical practice and research. Each item describes a specific symptom experienced over the past two weeks and is rated on a 0-to-3-point scale based on the option selected by the respondent. The global score of the test is obtained by directly summing the scores of all items, ranging from a minimum of 0 to a maximum of 63 points, where higher scores indicate greater symptom severity. To interpret this result, four standardized cut-off points are used to classify depression severity: a score of 0–13 points indicates minimal or absent depression; 14–19 points represents mild depression; 20–28 points corresponds to moderate depression; and 29–63 points indicates severe depression. Its psychometric adequacy and robustness in Spanish-speaking environments are supported by its corresponding Spanish validation (38).

The State-Trait Anxiety Inventory (STAI-Y) is a self-administered instrument designed to assess and distinguish between two independent dimensions of anxiety: state anxiety (a transient, fluctuating emotional state conditioned by the present moment) and trait anxiety (a latent, relatively stable propensity characteristic of the individual's personality). The inventory consists of 40 items in total, divided equally into two independent subscales of 20 statements each, with responses rated on a Likert-type intensity scale ranging from 0 to 3 points (or traditionally coded from 1 to 4 depending on the commercial version). The global score range for each subscale is calculated separately and usually ranges from 0 to 60 points (or from 20 to 80 points if the 1–4 format is applied), with higher scores indicating a higher level of anxiety. In the STAI-Y questionnaire, there are no absolute or fixed raw cut-off scores to categorize the condition; instead, interpretation is carried out by converting the raw scores obtained by the subject into standardized percentiles or centiles derived from specific normative data according to sex, age range, and the nature of the population (general or clinical). Generally, raw scores falling above the 75th percentile are interpreted as a high or clinically relevant level of anxiety. Its applicability and properties in the Hispanic population are supported by its corresponding Spanish validation citation (39).

##### 2.5.2.5 Other migraine-triggering factors

During the assessment visits, other potential triggers of acute migraine attacks, such as the menstrual cycle, stressors, or specific dietary components, will be recorded. In accordance with recent evidence emphasizing the predictive role of daily modifiable lifestyle factors in migraine occurrence (40), potential triggers will be prospectively logged in the daily headache diary. These include psychological stress, sleep disturbances, dietary factors, physical activity variations, and hormonal changes (menstrual cycle phase). Systematically capturing these daily lifestyle dynamics provides necessary context regarding individual attack susceptibility and potential baseline variability.

#### 2.5.3 Safety monitoring and adverse event reporting

Adverse Events (AEs) will be systematically monitored, recorded, and reported throughout the 24-week study period. Given the nature of the intervention, monitoring will focus on both general clinical AEs and potential intervention-specific side effects, including:

1. *Headache Exacerbation:* Transient worsening of migraine frequency or pain intensity during or following training sessions;
2. *Meditation-Related Distress:* Transient psychological discomfort, heightened anxiety, emotional distress, or restlessness during mindfulness practice;
3. *Device-Related Discomfort:* Skin irritation, pressure soreness, or physical discomfort caused by the Muse® headband dry sensors.

Participants will be instructed to log any unexpected symptoms or discomfort in their daily headache diary. Additionally, unprompted and spontaneously reported AEs will be systematically screened and recorded by clinical investigators during scheduled assessment visits (12 and 24 weeks). All reported AEs will be categorized according to severity (mild, moderate, severe) and evaluated for potential causal relationship to the NAMTP intervention. Any serious adverse event (SAE) will be reported immediately to the Drug Research Ethics Committee (CEIm) within 24 h. Participants experiencing persistent distress or severe discomfort will be offered clinical support and reserved the right to pause or discontinue intervention practice without affecting their standard routine care.

### 2.6 Statistical analysis

Data analysis will be conducted using IBM SPSS Statistics (v. 29.0), with a significance level of *α* = 0.05.

1. Initial Exploratory Analysis: Descriptive statistics (means and standard deviations for symmetric variables; medians and ranges for asymmetric variables; and frequencies for categorical variables) and visualization techniques will be applied. In accordance with CONSORT/SPIRIT guidelines, baseline demographic and clinical characteristics between groups will be presented using descriptive statistics without hypothesis testing (*p*-values). A bivariate analysis will subsequently be conducted to evaluate the preliminary relationship between key predictor variables (such as adherence) and the primary/secondary outcomes.
2. Longitudinal Statistical Modeling: After verifying the assumptions of normality of residuals and homoscedasticity, Linear Mixed Models (LMM) will be used to evaluate the effect of the intervention over time (baseline, 12, and 24 weeks) on the primary outcome (migraine frequency and severity) and secondary outcomes (pain, disability, anxiety, depression, and sleep). To evaluate the potential confounding impact of daily lifestyle triggers on headache frequency, reported triggers (such as perceived stress and sleep quality) will be summarized descriptively. In secondary exploratory analyses, key time-varying triggers will be included as covariates within the Linear Mixed Models (LMM) to evaluate their modulating effect on changes in MMDs across treatment arms.

LMMs were selected for their optimal capability to handle longitudinal missing data under the Missing At Random (MAR) assumption without requiring artificial imputation. The model will include “Group”, “Time”, and the Group Time interaction as fixed effects. The level of adherence will be entered as a covariate to assess its modulating effect, and participants will be treated as random effects (random intercepts). Intervention efficacy will be determined based on the fixed-effects coefficients. Null Hypothesis Significance Testing (NHST) and formal *p*-values will be primarily interpreted for the powered primary outcome (Monthly Migraine Days). For non-powered secondary outcomes, analysis will emphasize point estimations, 95% Confidence Intervals (95% CIs), and effect sizes (estimated using Cohen's *d*) to assess clinical relevance rather than relying strictly on statistical significance.

In accordance with the ICH E9 (R1) guideline on estimands and sensitivity analysis in clinical trials, major changes in preventive migraine medication (defined as the initiation of a new preventive class or a >50% dose modification) will be handled as intercurrent events. The primary estimand will reflect the treatment effect using an Intention-To-Treat (ITT) approach. To evaluate the robustness of the primary outcome against potential pharmacological confounding, a prespecified per-protocol (PP) sensitivity analysis will be conducted, excluding post-modification data points for participants experiencing major preventive treatment adjustments during the 24-week study period.

### 2.7 Ethical considerations

This study protocol was reviewed and formally approved by the Drug Research Ethics Committee (*Comité de Ética de la Investigación con Medicamentos*—CEIm) of the Salamanca Health Area (Spain) on July 30, 2025 (Ref. ID: 2025 07 1951). The study will be conducted in full compliance with the Declaration of Helsinki, the Oviedo Convention, and national regulations governing biomedical research. Participant safety, adverse event recording (including transient headache exacerbation, device discomfort, or meditation-related distress), and protocol compliance will be continuously overseen by the principal investigators in accordance with CEIm requirements.

Prior to initiating any study-related procedures, a trained clinical investigator will verbally explain the study objectives, procedures, potential risks, and benefits to all prospective participants. Candidates will be provided with a written Information Sheet and given sufficient time to ask questions. Written Informed Consent will be obtained from all participants before baseline evaluation. As this protocol does not involve ancillary studies or biological specimen collection, additional consent provisions for secondary use of biological samples are not applicable.

## 3 Discussion

This protocol describes a randomized controlled trial designed to further investigate the therapeutic potential of neurofeedback in adults with medium-to-high-frequency episodic migraine. Although neurofeedback has shown promising results in reducing the migraine-associated burden, the available evidence remains limited by significant methodological challenges, including study design heterogeneity, small sample sizes, variability in intervention protocols, and inconsistencies in outcome assessment strategies (14). In this context, the present study aims to contribute to the development of more standardized and clinically applicable evidence regarding the role of neurofeedback in migraine management.

Within this framework, it is hypothesized that the modulation of cortical activity through neurofeedback could help reduce the clinical burden of migraine. Consequently, the intervention could facilitate a decrease in the frequency and intensity of attacks, as well as improvements in associated disability and patients' quality of life. Furthermore, this study focuses on patients with medium-to-high-frequency episodic migraine, a population of high clinical relevance due to their elevated risk of functional impairment and progression toward chronic forms of the disease.

Given the multidimensional nature of migraine, assessing attack frequency alone is insufficient to capture the global therapeutic impact. As shown by Wells et al. (19), behavioral mindfulness interventions often exert their primary therapeutic value on functional disability, pain coping, and emotional well-being rather than direct reductions in headache days alone. By incorporating comprehensive measures of disability (MIDAS), headache impact (HIT-6), sleep, and affective distress, this trial will evaluate whether the additive effect of EEG neurofeedback within the NAMTP package can translate into broader clinical efficacy across both headache frequency and secondary quality-of-life parameters.

A critical aspect of non-pharmacological interventions in chronic pain management is the durability of therapeutic gains beyond the active treatment window. In this protocol, the active NAMTP intervention concludes at week 12. The subsequent assessment at week 24 represents a 12-week treatment-free follow-up period specifically designed to evaluate the retention and long-term sustainability of clinical improvements—such as reductions in Monthly Migraine Days (MMDs) and self-regulatory skills—once active neurofeedback feedback is removed. Assessing whether participants maintain therapeutic progress without ongoing device guidance will provide key insights into the potential lasting neurobehavioral consolidation induced by the intervention.

Although the neurobiological mechanisms underlying neurofeedback (NF) have not been fully elucidated, the available evidence suggests that its effects may be mediated by functional neuroplasticity processes capable of inducing both short- and long-term modifications in brain bioelectrical activity (41). These changes would involve the dynamic reorganization of interconnected cortical and subcortical networks related to sensory processing, emotional regulation, attentional control, and autonomic modulation (42). Since several of these subcortical brain networks are known to be dysfunctional in migraine, there is a biological rationale for utilizing NF in migraine therapy (43).

Nonetheless, several challenges must be considered when interpreting the potential implications of this protocol. First, a key limitation of this study is its open-label design; blinding participants to a behavioral neurofeedback intervention is unfeasible without a sham-device control. Consequently, non-specific treatment effects, participant expectations, or placebo responses cannot be completely ruled out, although standardized outcome assessments and blinded data analysis were implemented to minimize bias. When interpreting the outcomes of this study, it is important to emphasize that observed differences between groups reflect the overall pragmatic effect of the complete, multicomponent NAMTP package rather than isolated neurophysiological mechanisms of cortical self-regulation. While EEG neurofeedback serves as a core technological component, its therapeutic contribution is integrated with mindfulness practices, structured cognitive training, and continuous app-assisted engagement. Therefore, clinical improvements should be understood as the synergistic result of this multimodal digital neurobehavioral approach. Adherence to neurofeedback-based interventions can also represent a significant limitation, as they typically require sustained commitment, multiple training sessions, and active patient engagement. Furthermore, uncertainties persist regarding the optimal intervention protocols, training parameters, and neurophysiological targets most closely associated with clinical improvement in migraine. The literature also exhibits substantial heterogeneity in the EEG frequencies utilized, session duration and number, as well as application contexts, thereby hindering cross-study comparisons and the formulation of robust clinical recommendations. This study relies on a commercially available dry-sensor headband (Muse®) primarily as a behavioral and neurofeedback-assisted mindfulness tool rather than a diagnostic neurophysiological instrument. Due to budget constraints and logistical scope, formal quantitative electroencephalography (qEEG) or high-density cortical activity mapping at baseline and post-intervention was not included in the design. Consequently, direct physiological changes in specific EEG frequency bands (such as spectral alpha power shifts across sessions) will not be extracted for mechanistic mapping, and the device's proprietary “calm” score relies on internal algorithmic outputs rather than primary amplitude spectral analysis. Finally, due to the open-label pragmatic comparison against usual care alone, this trial cannot isolate the specific therapeutic effect of EEG neurofeedback from other program components (e.g., mindfulness practice or enhanced digital tracking). While this design maximizes ecological validity and reflects real-world clinical utility, future component-dismantling or sham-controlled studies will be necessary to determine the specific mechanistic impact of cortical self-regulation independently.

On the other hand, future research should address cost-effectiveness to evaluate its feasibility in clinical practice. Finally, the multidimensional and heterogeneous nature of migraine poses an additional methodological challenge, given that its clinical course and treatment response can be influenced by multiple biological, psychological, and contextual factors that are difficult to fully control in experimental settings.

In summary, this protocol addresses a question of growing clinical interest by evaluating the potential of EEG-based neurofeedback as a complementary treatment in patients with medium-to-high-frequency episodic migraine. Through a randomized controlled design and an assessment that incorporates multiple dimensions of the disease, the study aims to generate evidence that contributes to expanding knowledge on the therapeutic potential of neurofeedback and guiding the development of future non-pharmacological strategies for migraine management.
