# Supplementary material for: Effect of neurofeedback-assisted mindfulness training on migraine severity, associated disability, sleep quality, and emotional distress: protocol for a randomized controlled trial
Source: Front Digit Health. 2026 Oct 26;8:1962535. doi: 10.3389/fdgth.2026.1962535 (PMC13745676; doi:10.3389/fdgth.2026.1962535)
Supplement: Supplementary file 1 [file Table1.docx]

**Table S1**. Description of the Neurofeedback-Assisted Mindfulness Training Program (NAMTP)

| NUMBER OF THE SESSION | NAME OF THE SESSION | FOCUS | DURATION |
| --- | --- | --- | --- |
| 1 | Your First Mind Meditation | Discover Mind Biofeedback | 05:19 |
|  | Posture and Body Positioning | Discover Mind Biofeedback | 09:19 |
| 2 | Visualize your Brainwaves | Featured | 20:00 |
| 3 | River and Music | Browse by Goal: Rise Above Stress | 20:00 |
| 4 | Night Crickets Music | Browse by Goal: Rise Above Stress | 20:00 |
| 5 | Fresh Air: 4s-in 6s-out | Browse by Goal: Rise Above Stress | 20:00 |
| 6 | Resilence Refuge | Browse by Goal: Rise Above Stress | 11:21 |
|  | Bounce back from Adversity |  | 09:42 |
| 7 | Healing Drum | Browse by Goal: Elevate your performance | 20:00 |
| 8 | Gentle Rain | Browse by Goal: Elevate your performance | 20:00 |
| 9 | Rainforest | Browse by Goal: Improve Your Focus | 20:00 |
| 10 | Beach | Browse by Goal: Improve Your Focus | 20:00 |
| 11 | Oceanic Harmony 3D | Music | 10:00 |
|  | Zen Garden 3D |  | 10:00 |
| 12 | Northern Lights Serenade 3D | Music | 10:00 |
|  | Desert Mirage 3D |  | 10:00 |
| 13 | City Park | Mind | 20:00 |
| 14 | Ambient Music | Mind | 20:00 |
| 15 | Campfire and Music | Mind | 20:00 |
| 16 | Ocean and Music | Mind | 20:00 |
| 17 | Just birds | Mind | 20:00 |
| 18 | Wind Chimes | Posture | 20:00 |
| 19 | Crystal Cavern Music | Posture | 20:00 |
| 20 | River and Music | Browse by Goal: Rise Above Stress | 20:00 |
| 21 | Night Crickets Music | Browse by Goal: Rise Above Stress | 20:00 |
| 22 | Fresh Air: 4s-in 6s-out | Browse by Goal: Rise Above Stress | 20:00 |
| 23 | Resilence Refuge | Browse by Goal: Rise Above Stress | 11:21 |
|  | Bounce back from Adversity |  | 09:42 |
| 24 | Healing Drum | Browse by Goal: Elevate your performance | 20:00 |
| 25 | Gentle Rain | Browse by Goal: Elevate your performance | 20:00 |
| 26 | Rainforest | Browse by Goal: Improve Your Focus | 20:00 |
| 27 | Beach | Browse by Goal: Improve Your Focus | 20:00 |
| 28 | Oceanic Harmony 3D | Music | 10:00 |
|  | Zen Garden 3D |  | 10:00 |
| 29 | Northern Lights Serenade 3D | Music | 10:00 |
|  | Desert Mirage 3D |  | 10:00 |
| 30 | City Park | Mind | 20:00 |
| 31 | Ambient Music | Mind | 20:00 |
| 32 | Campfire and Music | Mind | 20:00 |
| 33 | Ocean and Music | Mind | 20:00 |
| 34 | Just birds | Mind | 20:00 |
| 35 | Wind Chimes | Posture | 20:00 |
| 36 | Crystal Cavern Music | Posture | 20:00 |
| 37 | River and Music | Browse by Goal: Rise Above Stress | 20:00 |
| 38 | Night Crickets Music | Browse by Goal: Rise Above Stress | 20:00 |
| 39 | Fresh Air: 4s-in 6s-out | Browse by Goal: Rise Above Stress | 20:00 |
| 40 | Resilence Refuge | Browse by Goal: Rise Above Stress | 11:21 |
|  | Bounce back from Adversity |  | 09:42 |
| 41 | Healing Drum | Browse by Goal: Elevate your performance | 20:00 |
| 42 | Gentle Rain | Browse by Goal: Elevate your performance | 20:00 |
| 43 | Rainforest | Browse by Goal: Improve Your Focus | 20:00 |
| 44 | Beach | Browse by Goal: Improve Your Focus | 20:00 |
| 45 | Oceanic Harmony 3D | Music | 10:00 |
|  | Zen Garden 3D |  | 10:00 |
| 46 | Northern Lights Serenade 3D | Music | 10:00 |
|  | Desert Mirage 3D |  | 10:00 |
| 47 | City Park | Mind | 20:00 |
| 48 | Ambient Music | Mind | 20:00 |
| 49 | Campfire and Music | Mind | 20:00 |
| 50 | Ocean and Music | Mind | 20:00 |
| 51 | Just birds | Mind | 20:00 |
| 52 | Wind Chimes | Posture | 20:00 |
| 53 | Crystal Cavern Music | Posture | 20:00 |
| 54 | River and Music | Browse by Goal: Rise Above Stress | 20:00 |
| 55 | Night Crickets Music | Browse by Goal: Rise Above Stress | 20:00 |
| 56 | Fresh Air: 4s-in 6s-out | Browse by Goal: Rise Above Stress | 20:00 |
| 57 | Resilence Refuge | Browse by Goal: Rise Above Stress | 11:21 |
|  | Bounce back from Adversity |  | 09:42 |
| 58 | Healing Drum | Browse by Goal: Elevate your performance | 20:00 |
| 59 | Gentle Rain | Browse by Goal: Elevate your performance | 20:00 |
| 60 | Rainforest | Browse by Goal: Improve Your Focus | 20:00 |
