# Supplementary material for: Effect of neurofeedback-assisted mindfulness training on migraine severity, associated disability, sleep quality, and emotional distress: protocol for a randomized controlled trial
Source: Front Digit Health. 2026 Oct 26;8:1962535. doi: 10.3389/fdgth.2026.1962535 (PMC13745676; doi:10.3389/fdgth.2026.1962535)
Supplement: Supplementary file 2 [file Table2.docx]

| **Table S2.** Record form for each session | | | | | |
| --- | --- | --- | --- | --- | --- |
| Number of the session: | | Name of the session: | | Type of session:  □ Guided □ Non-Guided | |
| Data: | | | Duration: | | |
| **MIND** | | | | | |
| % Calm: | Active mind time: | | Neutral mind time: | | Calm mind time: |
| **STILLNESS** | | | | | |
| % Still: | Active time: | | | Relaxed time: | |
| **HEART RATE** | | | | | |
| Average Heart rate: | High Heart rate: | | | Low Heart rate: | |
| **BREATHING** | | | | | |
| % High harmony: | Time low harmony: | | Time media harmony: | Time high harmony: | |
| Nº recoveries: | | | Nº birds: | | |
| *¿How did you feel during the session?*    Very stressed Stressed Neutral Relaxed Very relaxed | | | | | |
